# 18069 WISE Indiana (Wellbeing Informed by Science and Evidence in Indiana) - A state-university partnership response to the pandemic

**DOI:** 10.1017/cts.2021.765

**Published:** 2021-03-30

**Authors:** Amy Gilbert, Sarah Wiehe, Emily Hardwick, Amber Osterholt, Aaron Zych, Jennifer Sullivan

**Affiliations:** 1 Indiana Family Social Services Administration; 2 IU School of Medicine - Indiana Clinical & Translational Sciences Institute

## Abstract

ABSTRACT IMPACT: The WISE Indiana COVID-19 project facilitates rapid response and access to relevant and emerging evidence-based information for state personnel, healthcare providers and systems, managed care entities, community organizations, and all others involved in a professional capacity with the pandemic response. OBJECTIVES/GOALS: The COVID-19 project was developed to assist in responding to the Indiana Department of Health’s need for rapid and evidence-informed responses to complex questions about the pandemic and best practices for preventing, mitigating, monitoring and recovering from the COVID-19 global pandemic. METHODS/STUDY POPULATION: The WISE Indiana team was activated to assist in managing the project and immediately connected with university research librarians. Through our established networks, we were able to quickly engage academic researchers and clinicians across the state to rapidly respond to key questions about COVID-19 from government leadership. Research librarians added their expertise by conducting comprehensive searches of evidence-based clinical, public health, policy, and law literature and writing up detailed annotated bibliographies. Academic experts were also recruited to write daily summaries of emerging COVID-19 literature for the benefit of Indiana’s frontline responders and build and maintain an online repository of evidence-based learning materials for practitioners on the front lines. RESULTS/ANTICIPATED RESULTS: This work has informed key decision-making at many levels of Indiana’s COVID-19 response. Examples include data modeling for the IN.gov COVID-19 Dashboard, the allocation of Remdesivir, decisions about resuming elective procedures, and strategies for scaling back mitigation efforts. The WISE Indiana team has been able to engage over 40 academic experts from across the state of Indiana with expertise in pulmonary, infectious disease, law, epidemiology, mental health, public health, policy, and communications to assist in responding to key questions posed by government leadership and writing summaries of emerging COVID-19 literature which is summarized and accessible through our website: https://indianactsi.org/community/monon-collaborative/covid-19/. DISCUSSION/SIGNIFICANCE OF FINDINGS: The bidirectional exchange of information through the WISE Indiana collaborative network enable our team to quickly pivot to respond to the needs of our government leadership. Our team was able to rapidly translate the evidence-based information in order to respond to the policy and health outcomes needs of the state’s response to the global pandemic.

